# Integrated analysis of ceRNA network reveals potential prognostic *Hint1*-related lncRNAs involved in hepatocellular carcinoma progression

**DOI:** 10.1186/s12957-022-02535-z

**Published:** 2022-03-03

**Authors:** Cheng Zhang, Tianhao Bao, Yang Ke, Xin Liu, Xinghong Wang, Weiran Liao, Yutao He, Lin Wang

**Affiliations:** 1 Department of Hepatobiliary Surgery, The Second Affiliated Hospital of Kunming Medical University, No. 1168 Chunrongxi Road, Chenggong District, Kunming, Yunnan Province China; 2 Department of Hepatobiliary Surgery, The Second People’s Hospital of Chengdu, Chengdu, China; 3 Mental Health Center of Kunming Medical University, Kunming, China; 4 Department of Dermatology, The Second Affiliated Hospital of Chengdu Medical College, Chengdu, China

**Keywords:** *Hint1*, ceRNA, Hepatocellular carcinoma, lncRNA, Risk score

## Abstract

**Background:**

*Hint1* is a novel tumor suppressor gene, and inactivation of its expression is closely associated with the carcinogenesis of a variety of malignancies. The effects of *Hint1* deficiency on the competing endogenous RNA (ceRNA) regulatory network in the context of HCC remains to be fully characterized. This study aims to explore *Hint1*-related hub lncRNAs in HCC and to establish a reliable prognostic model for HCC patients based on these hub lncRNAs.

**Methods:**

lncRNA + mRNA microarray was used to identify differentially expressed (DE) lncRNAs and mRNAs in Huh7 cells before and after *Hint1* knockdown. A *Hint1*-related ceRNA network was mapped by bioinformation technology. The DEmRNAs in the network were analyzed via GO and KEGG enrichment analyses. Hub DElncRNAs associated with HCC patient prognosis were then detected through univariate and multivariate Cox regression analyses and were incorporated into a prognostic model. The prognostic value of this model was then assessed through the use of Kaplan-Meier curves, time-related ROC analyses, and nomograms. We also utilized Kaplan-Meier curves to validate the relationship between hub lncRNAs and the overall survival (OS) of HCC patients. Finally, A *Hint1*-related core ceRNA network based on the hub DElncRNAs and DEmRNAs was mapped.

**Results:**

We identified 417 differentially expressed DElncRNAs and 2096 DEmRNAs in Huh7 cells before and after *Hint1* knockdown. Three hub DElncRNAs (LINC00324, SNHG3, and DIO3OS) in the *Hint1*-associated ceRNA network were screened out using univariate and multivariate Cox regression analyses. A hepatocellular carcinoma (HCC) prognostic risk-scoring model and nomogram were constructed using these three hub lncRNAs, and it was confirmed that the risk score of the model could be used as an independent predictor of HCC prognosis. A *Hint1*-related core ceRNA network based on the hub DElncRNAs and DEmRNAs was also mapped.

**Conclusion:**

We constructed a reliable prognostic model for HCC patients based on three *Hint1*-related hub lncRNAs, and we believe these three hub lncRNAs may play critical roles in hepatocarcinogenesis, and progression.

**Supplementary Information:**

The online version contains supplementary material available at 10.1186/s12957-022-02535-z.

## Introduction

Hepatocellular carcinoma (HCC) is one of the most common cancers worldwide [1]. More than 700,000 people are diagnosed with HCC each year, and about 600,000 people die due to HCC and related complications [2]. Although great progress has been made in the research and treatment of liver cancer and the 10-year survival rate after radical resection has been improved to about 30%, about 50–70% of patients with radical resection of small liver cancer still have recurrence and metastasis within 5 years [3]. Therefore, exploring the carcinogenesis and progression mechanism of HCC, finding an effective way to inhibit metastasis and recurrence of HCC, and investigating the predictive factors of postoperative HCC recurrence are key to further improving the survival rate of patients with liver cancer.

At one time, non-coding RNAs (ncRNAs) were considered transcriptional noise with no biological activity [4]. However, in recent years, it has become clear that ncRNAs play a critical role in many cellular processes, such as epigenetic regulation, chromosome remodeling, transcriptional regulation, and post-translation modification [5–7]. Its abnormal expression can lead to the disorder of important biological processes such as cell proliferation, apoptosis, and cell migration and invasion, which leads to malignant cell transformation and even tumorigenesis [8–10]. In these ncRNAs, microRNAs (miRNAs) and long non-coding RNAs (lncRNAs) have attracted attention [4]. lncRNAs can regulate the inhibitory effects of miRNAs on downstream target genes through a mechanism involving competing endogenous RNA (ceRNA) and then participate in the carcinogenesis and progression of HCC [11–13].

Histidine triad nucleotide-binding protein 1 (HINT1) is a tumor suppressor, which belongs to the triple histidine superfamily [14, 15]. The epigenetic inactivation of the *Hint1* gene is a common and potential early event in the carcinogenesis of HCC [16]. HINT1 protein can regulate the transcription of a variety of cancer-related genes by directly binding a variety of transcription factors, including AP1, MITF, and USF2, thus affecting the carcinogenesis and progression of tumors [14, 15, 17]. However, the effects of *Hint1* deficiency on the ceRNA regulatory network of tumor cells need to be clarified.

Therefore, in this study, we used siRNA to knock down the expression of *Hint1* in Huh7 cells, comprehensively analyzed differentially expressed (DE) lncRNAs and mRNAs before and after knockdown using bioinformatics technology, and predicted the possible ceRNA network. Then, combined with TCGA (The Cancer Genome Atlas) database, a DElncRNAs diagnosis model was constructed, and hub DElncRNAs that may be regulated by *Hint1* were screened out. These hub DElncRNAs may help further clarify the molecular mechanism underlying the promotion of HCC progression by *Hint1*. We also expect these hub DElncRNAs to become promising biomarkers for predicting HCC progression and prognosis.

## Materials and methods

### siRNA transfection

*Hint1* siRNA (sc-92005) and interference control siRNA (sc-37007) were purchased from Santa Cruz Biotechnology (CA, USA). Transfection was performed with lipofectamine RNAiMax according to the manufacturer’s instructions.

### Total RNA extraction and quantitative real-time PCR

Total RNA was extracted as previously described [15]. Quantitative RT-PCR was performed using the SYBR green mix kit (Roche, Basel, Switzerland) and specific primers (Supplementary Table S1) in the ABI StepOne Plus real-time PCR system (Applied Biosystems, Foster City, CA, USA) in a volume of 20 μL. The relative quantification of mRNA levels was computed using the 2-^ΔΔCT^ method.

### Western blot

The relative levels of the target and control β-actin proteins were determined by Western blotting using cell lysates (30 μg/lane). Anti-HINT1 (ab124912, 1:1000) and anti-β-actin (ab8226, 1:500) were purchased from Abcam (Cambridge, MA, USA).

### Gene expression microarray profiling

TRIzol reagent was used to extract the total RNA of Huh7 cells treated with siHINT1 and siCTRL. The cDNA obtained by reverse transcription was hybridized to Agilent human lncRNA + mRNA Array V4.0(Boao, Beijing, China), which was designed with four identical arrays per slide (4×180K format) with each array containing probes interrogating about 41,000 human lncRNAs and about 34,000 human mRNAs. Microarray hybridization and computational analysis was performed as previously described [18].

### Construction of a ceRNA network

The possible target miRNA of DElncRNAs was predicted using the miRcode [19] database (http://www.mircode.org/). Three online databases including miRDB (http://mirdb.org), miRTarBase [20] (http://mirtarbase.mbc.nctu.edu.tw/php/index.php), and TargetScan [21] (http://www.ta-rgetscan.og) were used to search the targeted mRNAs of miRNAs. To improve the reliability of the results, only the miRNA-mRNA relational pairs found in all three databases were selected as candidate genes for the construction of ceRNA networks. Finally, Cytoscape software (version: 3.8.2) was used to visualize the ceRNA network based on the interactions among predicted DElncRNAs, predicted miRNAs, and predicted DEmRNAs [22].

### KEGG pathway and GO enrichment analyses

GO and KEGG enrichment analyses of DEmRNAs within the ceRNA network were performed using the “enrichgo” and “enrichkegg” functions within the “cluster profiler” package of the R software (version: 4.0.4); both *P* and *Q* values ≤ 0.05 were considered significantly enriched terms.

### Data from TCGA database

The RNA-sequencing (RNA-seq) data of clinical specimens and corresponding clinical data of hepatocellular carcinoma (LIHC) patients were downloaded from the TCGA database (https://portal.g-dc.cancer.gov/). The “limma” package of R software was used to normalize the RNA-seq data, and significantly differentially expressed genes with an average of less than 1 were excluded. Then, using the “createDataPartition” function in the R software “caret” package, the TCGA case cohort was randomly split (50:50%) into a training group and testing group for subsequent analysis.

### Construction of prognosis prediction model and nomogram

Using the “coxph” and “survdiff” functions of the “survival” package of R software, the survival correlated univariate Cox regression analysis of ceRNA related DElncRNA was carried out, and significant candidate hub DElncRNAs were screened out applying *P*<0.05 as a cutoff. Then, a multivariate stepwise Cox proportional risk regression model was constructed using the “caret,” “glmnet”, and “survminer” packages of R software, and the risk score model of each sample was obtained according to the following formula:

$$\mathrm{Risk}\ \mathrm{score}=\sum \left(\upbeta \ast \mathrm{ExpDElncRNAs}\right)$$

where *β* represents the coefficient (coef) of gene in the multivariate stepwise Cox analysis., and Exp represented the gene expression level. Then the receiver operating characteristic curve (ROC) of the training and testing groups was analyzed using the “survivalroc” package of R software to confirm and evaluate the predictive ability of the above models. Finally, the nomogram function of the “RMS” package of the R software was used to generate the nomogram.

### Validation of prognostic significance

The “Kaplan Meier plotter” [23] online tool (https://kmplot.com/analysis/) was used to verify the prognostic value of hub DElncRNAs in HCC patients.

### Statistical analysis

Numerical data are presented as mean ± standard error of the mean (SEM). Differences between groups were determined using a Student’s *t* test with a significance set of *P*≤0.05. Pearson’s chi-square tests were used to analyze the correlation between risk score and clinical features; the median risk score was used as the cutoff, and *P* ≤ 0.05 was considered significant.

## Results

### Knockdown of *Hint1* and microarray profiling

**Fig. 1:**
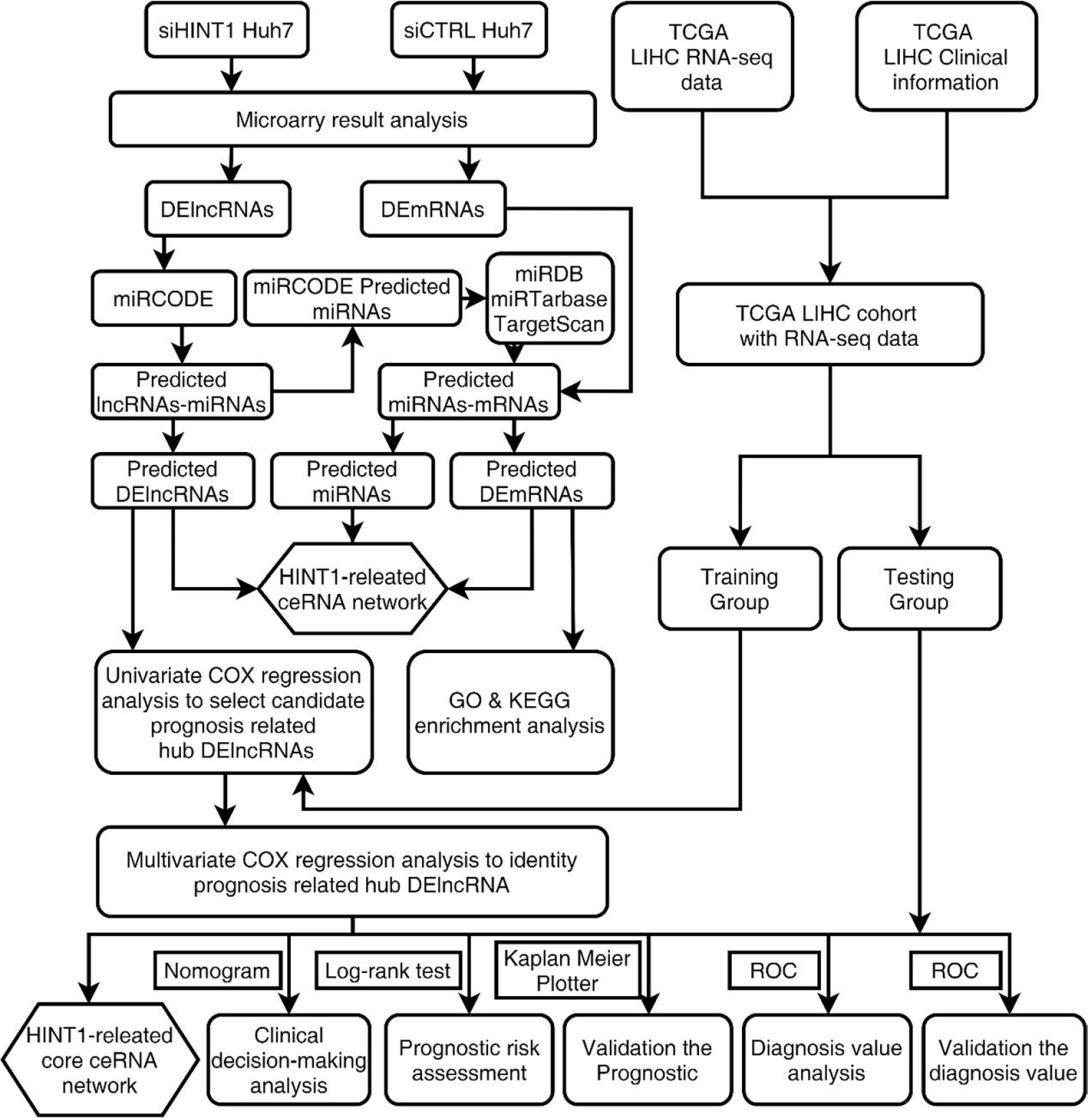
Flow chart of *Hint1*-related ceRNA network analysis. DElncRNAs, differentially expressed RNAs; DEmRNAs, differentially expressed mRNAs; ceRNA, competing endogenous RNA; GO, Gene Ontology; KEGG, Kyoto Encyclopaedia of Genes and Genomes; TCGA, The Cancer Genome Atlas; LIHC, liver hepatocellular carcinoma; RNA-seq, RNA-sequencing; ROC, receiver operating characteristic curve

**Fig. 2:**
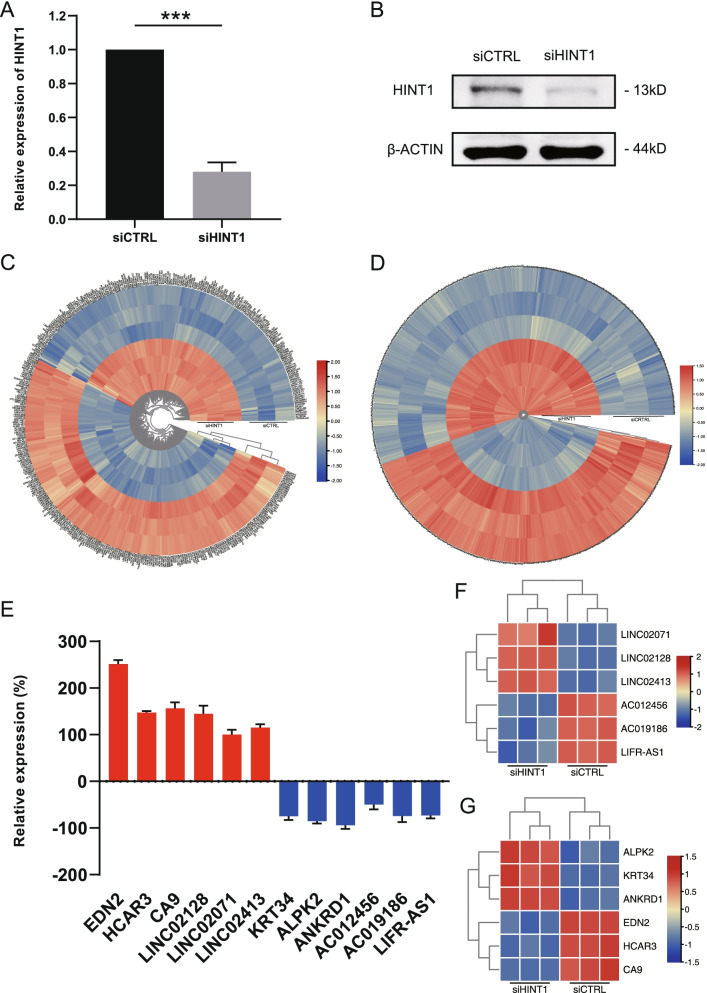
Microarray profiling and validation in *Hint1*-deficient Huh7 cells. **A** mRNA level of *Hint1* in both siCTRL- and siHINT1-treated Huh7 cells determined by qRT -PCR (normalized to the mRNA level of β-ACTIN, data was presented as mean ± SEM; *n* = 3). **B** Protein level of HINT1 and β-ACTIN in both siCTRL- and siHINT1-treated Huh7 cells determined by western blot. Duplicate lncRNAs (**C**) and mRNAs (**D**) expression microarray analysis of Huh7 cells treated with either siCTRL or siHINT1 were subjected to log2-transformed gene expression followed by K-means clustering (fold-change (FC) ≥ 2, while false discovery rate (FDR) ≤ 0.05, up- and downregulated lncRNAs and mRNAs are indicated in red and green, respectively). **E** qRT-PCR was performed on twelve lncRNAs and mRNAs with the highest levels of elevation and decrease in the microarray analysis and normalized to mRNA level of β-ACTIN (FCs were computed in terms of percentage fold change of the siHINT1-treated transcriptome relative to the siCTRL-treated transcriptome, data was presented as mean ± SEM; *n* = 3); Mini heat map representing the selected twelve lncRNA (**F**) and mRNA (**G**) in microarray analysis. ***P ≤ 0.001

The study design is illustrated in Fig. 1. siRNA was used for knockdown of *Hint1* expression in Huh7 cells. As shown in Fig. 2, the expression of *Hint1* decreased significantly by treatment of siHINT1. After confirming this knockdown effect, we ran a lncRNA + mRNA expression microarray with total RNA extracted from *Hint1*-deleted Huh7 cells (Huh7 cells treated with siCTRL as control). *Microarray raw data have been uploaded to the GEO (Gene Expression Omnibus) database (GEO Accession: GSE177624)*. After preliminary processing of the microarray data, we identified 417 DElncRNAs (231 were upregulated and 186 were downregulated, Fig. 2C) and 2096 DEmRNAs (899 were upregulated and 1197 were downregulated, Fig. 2D) with |FC|≥2 and FDR<0.05 as the cutoff values. We selected three lncRNAs with the most significant changes in expression, as well as mRNAs, for PCR verification. There was a good correlation with the microarray results (Fig. 2E–G).

### Prediction of ceRNA network regulated by *Hint1*

We first used the miRcode database to predict the potential miRNAs that may interact with DElncRNAs. We then utilized three online databases (miRDB, miRTarBase, and TargetScan) to predict the potential target mRNAs of miRNAs. Next, we intersected these predicted mRNAs with the DEmRNAs to obtain DEmRNAs that may be involved in the ceRNA network. We ultimately identified 135 predicted DElncRNAs and 185 predicted DEmRNAs that may have regulatory relationships, as well as 40 predicted miRNAs that may be involved in ceRNA networks. We visualized the final ceRNA network using Cytoscape software; the network included 360 nodes with 1738 interactions (Fig. S1).

### GO and KEGG enrichment analyses

The 185 predicted DEmRNAs that may be involved in pertinent biological processes and pathways were further investigated. Using the “Cluster Profiler” package of the R software, we carried out GO and KEGG enrichment analyses, and used the “Enrich Plot” package to visually display the results. The top 10 of 44 significant GO terms and the top 10 of 17 significant KEGG pathways are listed in Table 1. Of these terms, “RNA polymerase II transcription regulator complex,” “intrinsic apoptotic signaling pathway,” and “misfolded protein binding” were the top GO terms in the cellular component (CC), biological process (BP), and molecular function (MF) categories, respectively. “Kaposi sarcoma-associated herpesvirus infection,” “microRNAs in cancer,” “measles,” “p53 signaling pathway,” and “Pertussis” were the first five KEGG pathways.

**Table 1:** Top 10 significant GO and KEGG terms identified by enrichment analysis

Term		*P*-value	*Q*-value
GO Category			
CC	RNA polymerase II transcription regulator complex	1.63E-05	0.004217
BP	Intrinsic apoptotic signaling pathway	1.75E-05	0.029246
BP	Response to steroid hormone	2.16E-05	0.019874
BP	Cellular response to external stimulus	5.49E-05	0.041367
BP	Female pregnancy	9.17E-05	0.036427
BP	Muscle cell proliferation	9.65E-05	0.048769
MF	Misfolded protein binding	0.000116	0.437985
BP	Oligopeptide transport	0.000123	0.035732
BP	Response to nutrient levels	0.000138	0.044587
BP	Regulation of intrinsic apoptotic signaling pathway	0.000162	0.035867
KEGG pathway			
Kaposi sarcoma-associated herpesvirus infection		6.74E-05	0.012491
MicroRNAs in cancer		0.000795	0.034294
Measles		0.001041	0.024159
p53 signaling pathway		0.001451	0.044321
Pertussis		0.001736	0.034137
PI3K-Akt signaling pathway		0.002314	0.021461
Colorectal cancer		0.002992	0.043393
Lipid and atherosclerosis		0.003169	0.033353
Insulin signaling pathway		0.004785	0.028643
Yersinia infection		0.004834	0.034643

*GO* Gene Ontology, *CC* cellular component, *BP* biological process, *MF* molecular function, *KEGG* Kyoto Encyclopaedia of Genes and Genomes

### Screening of prognosis-related lncRNA

The RNA-seq data and their corresponding clinical data in the LIHC subset of the TCGA database were randomly divided into a training group (186 cases) and testing group (184 cases) for the following process. Considering that lncRNA was in the upstream part of the ceRNA network and it is the main effector of miRNAs and mRNAs, we carried out survival-related univariate Cox regression analysis of the 135 predicted DElncRNAs in the ceRNA network with the training group data. Six survival-related candidate hub DElncRNAs were screened out (Fig. S2A). Subsequently, these six candidate hub DElncRNAs were used to perform multiple stepwise Cox regression, to further investigate their potential effects on the survival time and clinical outcomes of patients. Finally, three hub DElncRNAs, LINC00324, SNHG3, and DIO3OS, were identified as survival-related independent predictors for HCC patients (Fig. S2B, Table 2).

**Table 2:** Three prognosis-associated hub DElncRNAs identified by stepwise multivariate Cox regression analysis in the training group

	Coef	HR	Lower 95% CI	Upper 95% CI	*P* value
LINC00324	− 0.232	0.793	0.570	0.951	0.039*
SNHG3	0.078	1.081	1.016	1.150	0.013*
DIO3OS	− 0.601	0.548	0.329	0.914	0.021*

*Coef* coefficient value, *HR* hazard rate, *CI* confidence interval. *P < 0.05

### Construction and analysis of prognosis-related lncRNA risk-scoring model

Next, we used the three hub lncRNAs and their coefficient values obtained in multiple stepwise Cox regression analysis to construct a multivariate Cox proportional hazards regression model, and calculated a risk score for each patient in the training group according to the following formula:

$$Risk\;score=\left(-0.2320\ast ExpLINC00324\right)+\left(0.0782\ast ExpSNHG3\right)+\left(-0.6007\ast ExpDIO3OS\right)$$

**Fig. 3:**
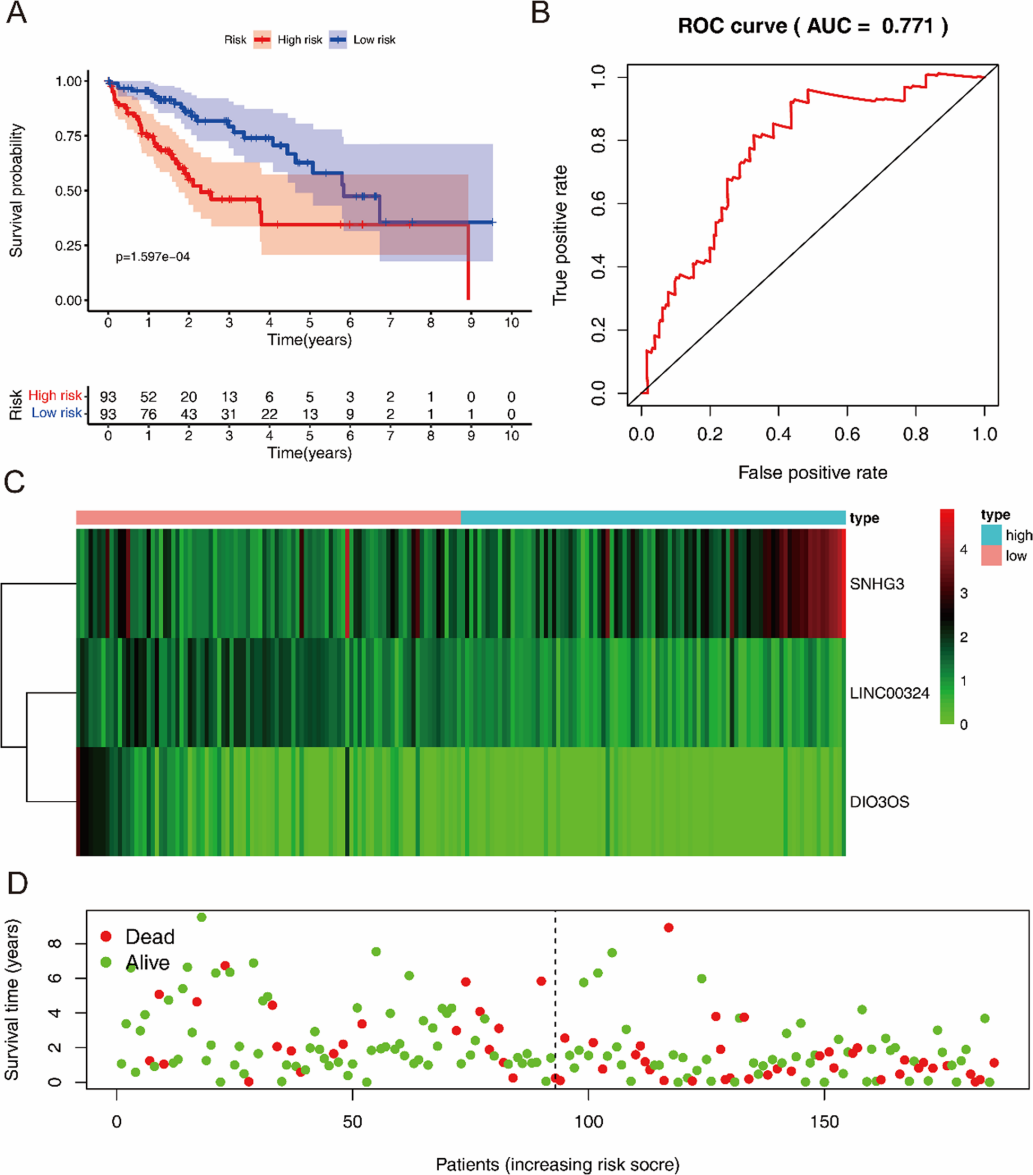
Risk score analysis of three-hub lncRNAs prognostic model in the training group of TCGA LIHC cohort (*n* = 186). **A** Survival curve for low- and high-risk subgroups. **B** ROC curves for forecasting OS based on risk score. **C** Expression heat map of the three-hub lncRNAs for low- and high-risk subgroups. **D** Risk score distribution, and the survival status for low- and high-risk subgroups

Furthermore, the training group was divided into low-risk and high-risk subgroups according to the median risk score. Then the differences in overall survival (OS) between these two subgroups were compared, the high-risk subgroup had a significantly OS than the low-risk subgroup (*P*<0.001, Fig. 3A). To further evaluate the utility of the three hub DElncRNAs as prognostic biomarkers, time-dependent ROC analysis was performed. The area under the ROC curve (AUC) of the lncRNA risk scoring model was 0.775, indicating moderate diagnostic performance (Fig. 3B–D).

**Fig. 4:**
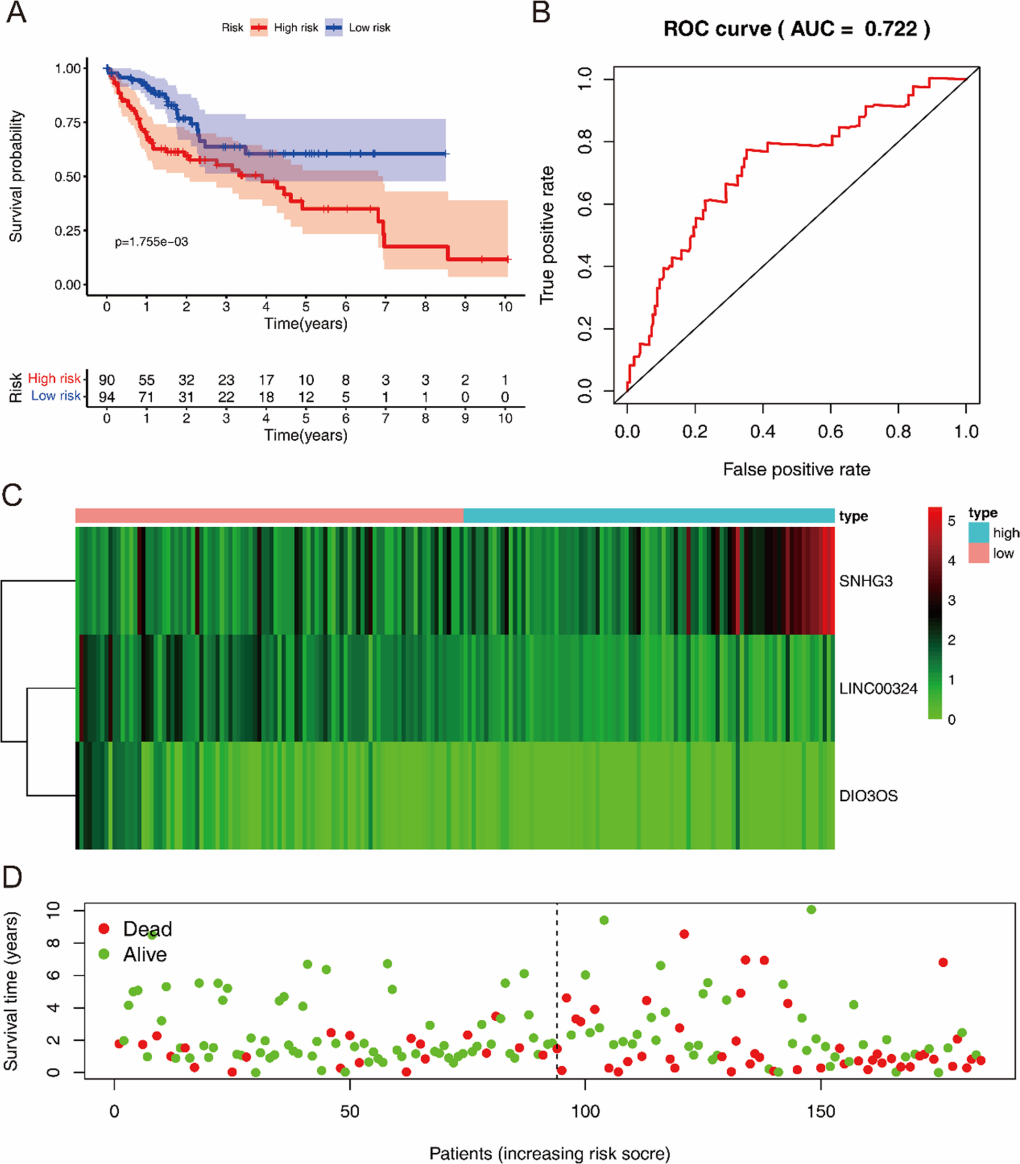
Risk score analysis of three-hub lncRNAs prognostic model in the testing group of TCGA LIHC cohort (*n* = 184). **A** Survival curve for low- and high-risk subgroups. **B** ROC curves for forecasting OS based on risk score. **C** Expression heat map of the three-hub lncRNAs for low- and high-risk subgroups. **D** Risk score distribution, and the survival status for low- and high-risk subgroups

In addition, we evaluated whether the risk-scoring model was against the testing cohort. Similar to the results for the training group, the OS of patients with a high risk score in the testing group was worse than that of patients with a low risk score (Fig. 4A). And the model had good sensitivity and specificity, with an AUC of 0.722 (Fig. 4B–D).

Meanwhile, we also analyzed the association between risk score and other clinicopathological parameters in the testing group. As was shown in Table S2, the level of risk score was correlated with tumor stage and tumor sizes.

### Nomogram drawn based on the three hub DElncRNAs

**Fig. 5:**
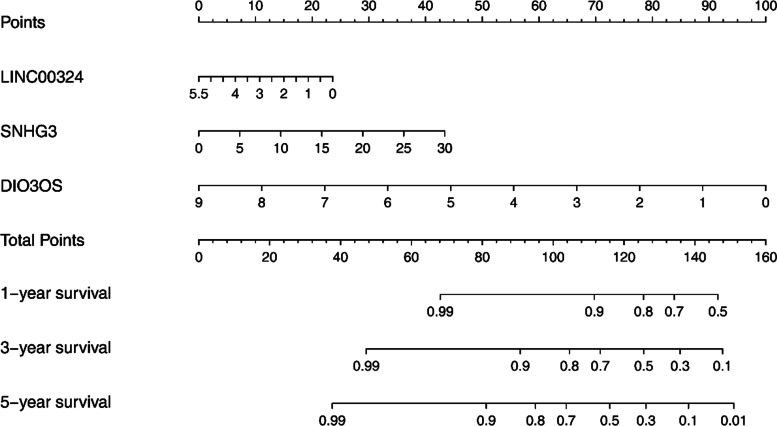
Nomogram for predicting 1-, 3-, and 5-year OS of LIHC patients in the TCGA cohort

To predict the prognosis of HCC patients more intuitively, the “rms” package of the R software was used to draw a nomogram based on the three hub DElncRNAs (Fig. 5). Using that nomogram, the estimated survival rate of patients at 1, 3, and 5 years could be obtained by drawing a vertical line between the total integral axis and each pre-posterior axis, which could help doctors make clinical decisions for HCC patients. In addition, the prognostic significance of different clinical features in the testing group was also evaluated using univariate Cox regression analysis. Tumor stage, tumor size, and risk score were correlated with OS in patients with HCC (Table 3); however, a multiple Cox regression analysis showed that risk score was the only independent prognostic factor related to OS (Table 3).

**Table 3:** The prognostic value of different clinical parameters in the testing group

	Univariate analysis			Multivariate analysis		
	HR	95% CI	*P* value	HR	95% CI	*P* value
Age	1.00	0.98–1.02	0.899	1.00	0.98–1.02	0.828
Gender	0.96	0.57–1.61	0.869	0.98	0.56–1.70	0.929
Stage	1.69	1.28–2.23	<0.001***	1.81	0.74–4.39	0.192
T	1.64	1.27–2.13	<0.001***	0.90	0.37–2.20	0.825
M	1.33	0.76–2.34	0.315	1.78	0.91–3.49	0.093
N	1.00	0.55–1.82	0.995	0.75	0.35–1.58	0.445
Risk score	1.12	1.04–1.21	0.002**	1.10	1.01–1.20	0.031*

*T* tumor size (> 5cm vs.≤ 5cm), *N* lymph node metastasis (yes vs. no), *M* distant migration (yes vs. no). *P < 0.05, **P < 0.01, ***P < 0.001

### Validation of the prognostic value of hub DElncRNAs

To further evaluate the prognostic value of the three hub lncRNAs in HCC, the “Kaplan Meier-plotter” online tool was used to analyze the relationship between each hub lncRNA and OS and draw Kaplan-Meier curves. All three hub lncRNAs were closely related to OS in HCC patients (Fig. S3).

### Core ceRNA network related to hub DElncRNAs

**Fig. 6:**
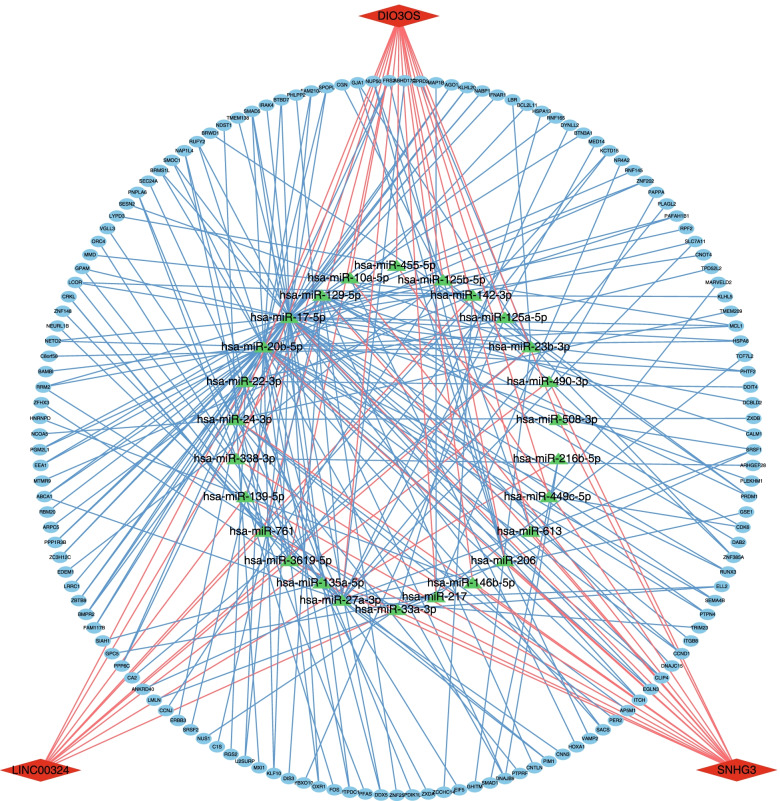
*Hint1*-related core ceRNA network created by hub DElncRNAs. Red diamonds represent 3 hub DElncRNAs (2.2% of all DElncRNAs); blue ellipses represent 143 DEmRNAs (77.3% of all DEmRNAs); yellow triangles represent 26 predicted miRNAs (65.0% of all predicted miRNAs)

Finally, on the basis of the original ceRNA network, Cytoscape software was used to screen and draw the core ceRNA network based on the three hub DElncRNAs. As shown in Fig. 6, the core network was composed of 172 nodes and 251 interactions, involving 143 DEmRNAs and 26 miRNAs that may participate in the network. This provides an important reference for further study of the specific mechanism of the ceRNA network regulated by *Hint1*.

## Discussion

HCC is the sixth most common malignant tumor worldwide [24]. Although hepatectomy, liver transplantation, and other therapies can improve the prognosis of HCC patients to a certain extent, it is still the third leading cause of cancer-related death in the world due to its high rate of invasion, metastasis, and postoperative recurrence [25]. Therefore, it is important to elucidate the molecular mechanism of the occurrence and progression of HCC and to find an effective target for its diagnosis and treatment. In recent years, with the development of high-throughput screening and bioinformatics technology, increasing numbers of lncRNAs have been found to be involved in gene regulation, particularly in ceRNA networks [26]. Meanwhile, the important role of ceRNA regulatory networks in the oncogenesis and progression of malignant tumors has gradually been recognized [27]. For example, lncRNA HIF1A-AS1 [28] and ZEB1-AS1 [29] were recently proved to be associated with HCC progression via ceRNA pattern, while lncRNA TTN-AS1 was demonstrated to act as a tumor promoter in gallbladder carcinoma by sponging miR-107 and upregulating HMGA1 [30].

*Hint1* is a newly discovered tumor suppressor gene. In 2003, Su et al. [31] first found that *Hint1* may have a tumor inhibitory effect by establishing a gene knockdown mouse model. Subsequent studies have found that its expression is decreased in many human malignant tumors, including HCC, non-small-cell lung cancer, and colon cancer [31–33]. HINT1 protein can affect the transcription of downstream target genes by interacting with a variety of transcription factors, thereby regulating the proliferation, apoptosis, invasion, and migration of tumor cells [34]. Specifically, our team found that *Hint1* can inhibit the proliferation of rectal cancer cells by directly binding to Posh-JNK2 complexes, inhibiting the transcriptional activity of AP-1, which is an important cancer transcription factor [15]. Motzik et al. [17] confirmed that HINT1 protein can promote apoptosis of melanoma cells by directly binding to the DNA binding protein MITF. Considering the role of *Hint1* in the regulation of transcription, we speculated that it may indirectly regulate the ceRNA network by affecting the transcription of lncRNAs, thus influencing the progress of malignant tumors. Therefore, in this study, we first used a lncRNA + mRNA microarray to detect the DElncRNAs and DEmRNAs in Huh7 cells before and after *Hint1* knockdown. This identified 417 DElncRNAs and 2096 DEmRNAs, from which we constructed the ceRNA network.

As the proteins translated by mRNA in ceRNA regulatory networks are the final functional implementers, GO and KEGG enrichment analyses were conducted on the DEmRNAs in the ceRNA network, to better understand how *Hint1*-related ceRNA networks may be involved in the carcinogenesis and progression of HCC. GO enrichment analysis showed that DEmRNAs in the network were mainly involved in “RNA polymerase II transcription regulator complex” terms, indicating that the transcriptional regulatory activity of *Hint1* may partly be based on the ceRNA network. In addition, DEmRNAs were highly enriched in “intrinsic apoptotic signaling pathway” terms, which to some extent explains the effects of *Hint1* on apoptosis of HCC cells reported by Hsieh et al. [35], namely, that *Hint1* may partly regulate the apoptosis of HCC cells by regulating the ceRNA network. In KEGG enrichment analysis, the DEmRNAs were mainly involved “microRNAs in cancer,” “p53 signaling pathway,” and other cancer-related pathways, which further confirmed that *Hint1*-related ceRNA regulatory networks played an important role in HCC.

lncRNAs are located in the upstream of ceRNA network and its expression level may be closely related to the prognosis of patients with malignant tumors [6]. Therefore, we used univariate and multivariate stepwise Cox regression analyses to find hub DElncRNAs related to the prognosis of HCC using the TCGA database. LINC00324, SNHG3, and DIO3OS were identified as survival-related hub lncRNAs. Among them, DIO3OS has been reported to inhibit the proliferation and invasion of HCC cells by competitive binding to miR-328 [36]; our results are consistent with that finding. In addition, Zhang et al. [37] reported that SNHG3 can promote the EMT process of HCC cells through competitive binding of miR-128, and then release CD151. However, there are no reports suggesting that LINC00324 is involved in the occurrence or development of HCC, although some studies have reported that it is a risk factor for disease progression in non-small-cell lung cancer and osteosarcoma [38, 39]. The functional differences of LINC00324 between different tissues is worth further investigating. Furthermore, we established a prognostic risk-scoring model of HCC based on three hub DElncRNAs using multivariate stepwise Cox regression analysis. The DElncRNAs had good diagnostic ability and could screen out HCC patients with a poor prognosis. Subsequently, we mapped a nomogram to help us more intuitively predict the OS of HCC patients for 1, 3, and 5 years. Validation tests indicated that all of them were closely related to OS in HCC patients. Finally, we constructed an *Hint1*-related core ceRNA network based on the three hub DElncRNAs. Interestingly, in this core network, these three hub DElncRNAs regulated 77% of the DEmRNAs through 24 miRNAs, confirming their importance in the network. Among these 24 miRNAs, miR-23b-3p was reported to be involved in the epithelial-mesenchymal transition (EMT) of HCC by targeting EOMES mRNA [40], and miR-10a-5p has also been proved to be associated with migration and invasion in HCC cells [41]. At the same time, the apoptosis-related gene *PIM1* [42], proliferation-related gene *CCND1* [43], migration- and invasion-related gene *BMPR2* [44], and tumor immunocyte infiltration-related gene *IFNAR1* also appeared in the core network [45]. This indicates the diversity of hub lncRNAs in the regulation of the tumor malignant phenotype, and also confirms the significant role of the *Hint1*-related ceRNA network in malignant tumors. Overall, our prognostic model is based on three lncRNAs, which significantly reduces the cost of clinical gene sequencing and is more favorable for clinical application. In addition, our prognostic model provided a complementary perspective on individual tumors and developed an individual scoring system for HCC patients, and the nomogram based on this scoring system may be a promising tool for clinicians in the future.

However, there were some limitations to this study. First, due to the lack of a liver cancer lncRNA database, our prognosis model is only based on data from the TCGA database and has not been verified in a clinical cohort or other databases. Second, although we used several online databases to predict the miRNAs involved in the ceRNA network, miRNAs expression needs to be validated in both in vivo and in vitro experiments.

## Conclusions

In conclusion, we systematically investigated the composition of the *Hint1*-related ceRNA network for HCC and the prognostic value of specific lncRNAs in HCC through a series of bioinformatics analyses. We identified three hub DElncRNAs related to patient prognosis, constructed an HCC prognostic risk score model with these three lncRNAs, and verified that the model could be used as an independent predictor of HCC prognosis. To the best of our knowledge, this is the first study to establish an HCC prognostic model for *Hint1*-related lncRNAs. Our results may contribute to further research into the molecular mechanisms underlying the promotion of HCC progression by *Hint1*, and we expect these three lncRNAs will become promising biomarkers for predicting HCC progression and prognosis.

## Supplementary Information

**Additional file 1: Table S1.** The sequences of primers for quantitative PCR (qPCR). **Table S2.** Correlation between risk score and other clinicopathological parameters in testing group.

**Additional file 2: Fig**ure **S1.** *Hint1*-related ceRNA network. Blue ellipses represent 185 DEmRNAs; red diamonds represent 135 DElncRNAs; yellow triangles represent 40 predicted miRNAs.

**Additional file 3: Figure S2.** (A) Univariate Cox regression analysis for identification of hub lncRNAs in the training group of TCGA LIHC cohort. (B) Multivariate Cox regression analysis to identify prognosis related hub lncRNAs in the training group of TCGA LIHC cohort.

**Additional file 4: Figure S3.** The expression of (A) DIO3OS (B) LINC00324 and (C) SNHG3 were associated with the OS in LIHC patients in Kaplan Meier Plotter server.

## Data Availability

*Microarray raw data have been uploaded to the GEO database (* https://www.ncbi.nlm.nih.gov/geo/query/acc.cgi?acc=GSE177624*).* The other data used or analyzed during the study are available from the corresponding author on reasonable request.
