# Supplementary material for: Integrated analysis of ceRNA network reveals potential prognostic Hint1-related lncRNAs involved in hepatocellular carcinoma progression
Source: World J Surg Oncol. 2022 Mar 3;20:67. doi: 10.1186/s12957-022-02535-z (PMC8896107; doi:10.1186/s12957-022-02535-z)
Supplement: Supplementary file 1 — Additional file 1: Table S1. The sequences of primers for quantitative PCR (qPCR). Table S2. Correlation between risk score and other clinicopathological parameters in testing group. [file 12957_2022_2535_MOESM1_ESM.docx]

**Supplementary Materials**

| Table S1: The sequences of primers for quantitative PCR (qPCR). | | |
| --- | --- | --- |
| Genes | Forward (5’-3’) | Reverse (5’- 3’) |
| *HINT1* | CTCAAGCACCAACACATT | CCAAGAAGACTTTCATCATCA |
| *EDN2* | GCCAGCGTCCTCATCTAT | GCCGTAAGGAGCTGTCTGTTC |
| *HCAR3* | CTGTTTCCACCTCAAGTCCTGG | CAGTCTGAACGCCGCACATAGT |
| *CA9* | GGGTGTCATCTGGACTGTGTT | CTTCTGTGCTGCCTTCTCATC |
| *LINC02128* | CAGCCTGACTCCCACTTCCAG | CCCAGTCACGGAGGTTTACCA |
| *LINC02071* | GCCAGCCTCAAGAGAGGAAGA | ATGGAAATGCAGAAATCACCCGT |
| *LINC02413* | AGGCTATGCAAAGAGGGGTAC | TCAGCAATTTGGGATAAACG |
| *KRT34* | AGACCAGGAGTCAGTATGAGGC | GCTGGATACCACCTGCTTGTTC |
| *ALPK2* | TCCGAAGGACCAGGGACTCTAT | CGGTGAACCCCTTCTCCAAA |
| *ANKRD1* | TCGTTCCAGCTGTGGTCCACA | CTTCTTCCAGTGACCAGTTCCTCT |
| *AC012456* | AACAGGCTCAAATTCAAGTC | CTGTAGAATGTGAGGATGGC |
| *AC019186* | TGAACATGCCGGAATGTCTT | GACCTTGACCACCCAACCAC |
| *LIFR-AS1* | GCAAATACTGTGTATTAGTCC | CCGCTTCCTTGTGAAGAAGGT |
| *β-actin* | CTCCATCCTGGCCTCGCTGT | GCTGTCACCTTCACCGTTCC |

| Table S2: Correlation between risk score and other clinicopathological parameters in testing group | | | | |
| --- | --- | --- | --- | --- |
| Clinicopathological parameters | Number of cases | Risk score | | *P*-value |
|  | (n=171) | High (n=85) | Low (n=86) |  |
| Age |  |  |  | 0.107 |
| <60 years | 82 | 35 | 47 |  |
| ≥60 years | 89 | 50 | 39 |  |
| Gender |  |  |  | 0.547 |
| Male | 116 | 60 | 56 |  |
| Female | 55 | 25 | 30 |  |
| Stage |  |  |  | 0.024* |
| I-II | 123 | 54 | 69 |  |
| III-IV | 48 | 31 | 17 |  |
| T |  |  |  | 0.033* |
| <5cm | 126 | 56 | 70 |  |
| ≥5cm | 45 | 29 | 16 |  |
| M |  |  |  | 0.165 |
| Yes | 41 | 16 | 25 |  |
| No | 130 | 69 | 61 |  |
| N |  |  |  | 1.000 |
| Yes | 39 | 19 | 20 |  |
| No | 132 | 66 | 66 |  |
| T, tumor size; N, lymph node metastasis; M, distant migration. The median level of risk score (risk score = 1.124) was used as the cutoff. *, *P*<0.05. | | | | |
