# Supplementary figures and images for: Integrated analysis of ceRNA network reveals potential prognostic Hint1-related lncRNAs involved in hepatocellular carcinoma progression
Source: World J Surg Oncol. 2022 Mar 3;20:67. doi: 10.1186/s12957-022-02535-z (PMC8896107; doi:10.1186/s12957-022-02535-z)

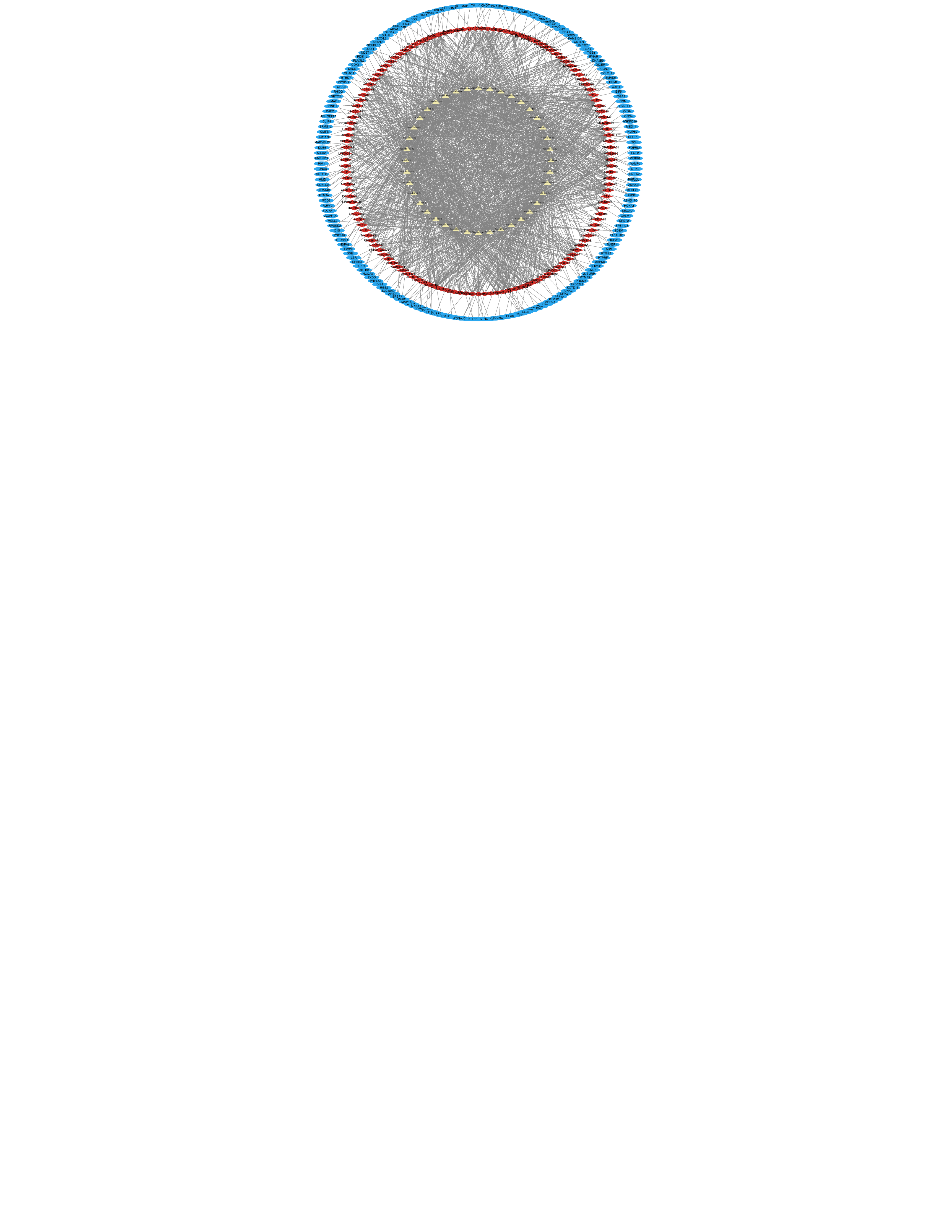

Supplement: Supplementary file 2 — Additional file 2: Figure S1. Hint1-related ceRNA network. Blue ellipses represent 185 DEmRNAs; red diamonds represent 135 DElncRNAs; yellow triangles represent 40 predicted miRNAs. [file 12957_2022_2535_MOESM2_ESM.tif]

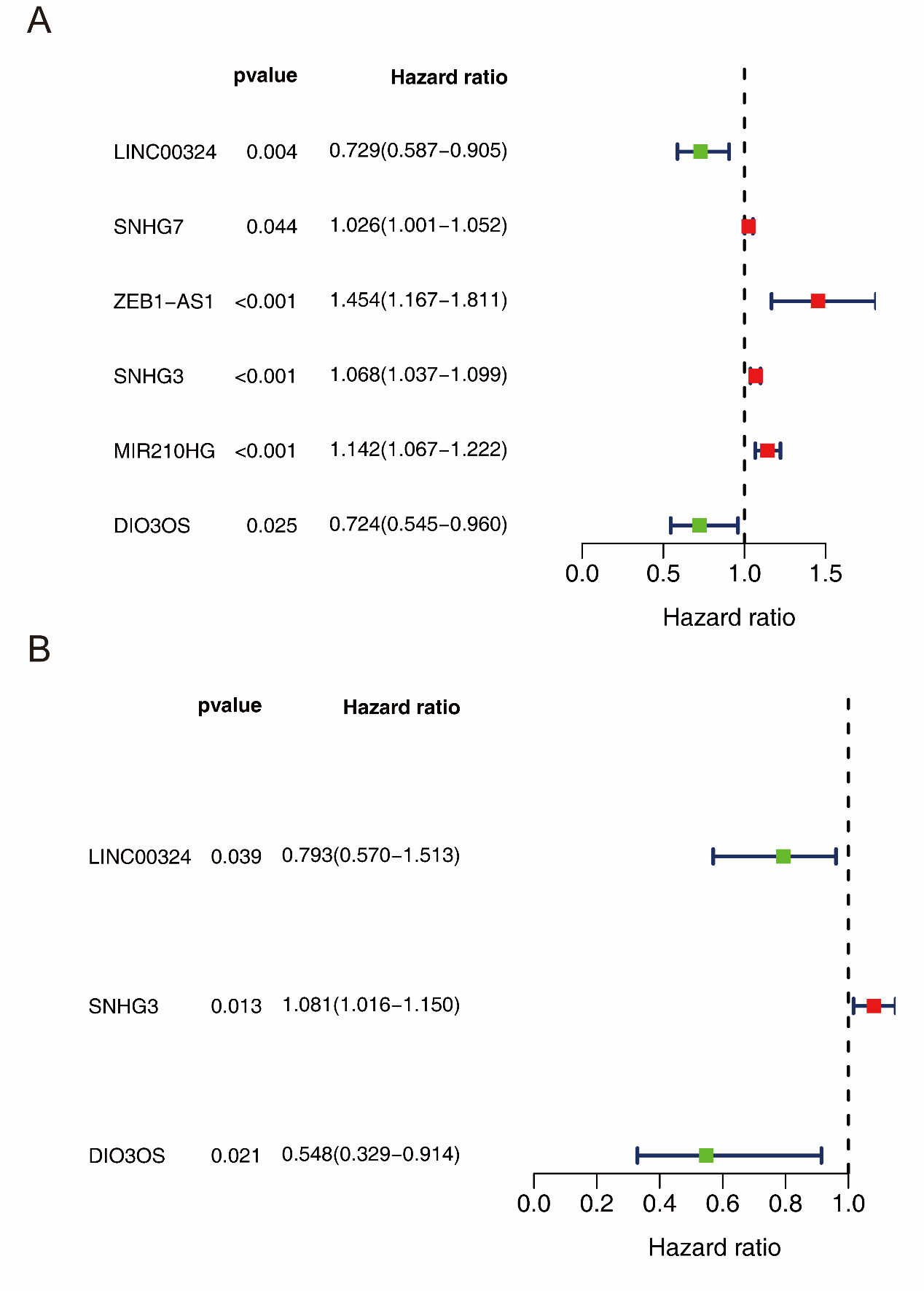

Supplement: Supplementary file 3 — Additional file 3: Figure S2. (A) Univariate Cox regression analysis for identification of hub lncRNAs in the training group of TCGA LIHC cohort. (B) Multivariate Cox regression analysis to identify prognosis related hub lncRNAs in the training group of TCGA LIHC cohort. [file 12957_2022_2535_MOESM3_ESM.tif]

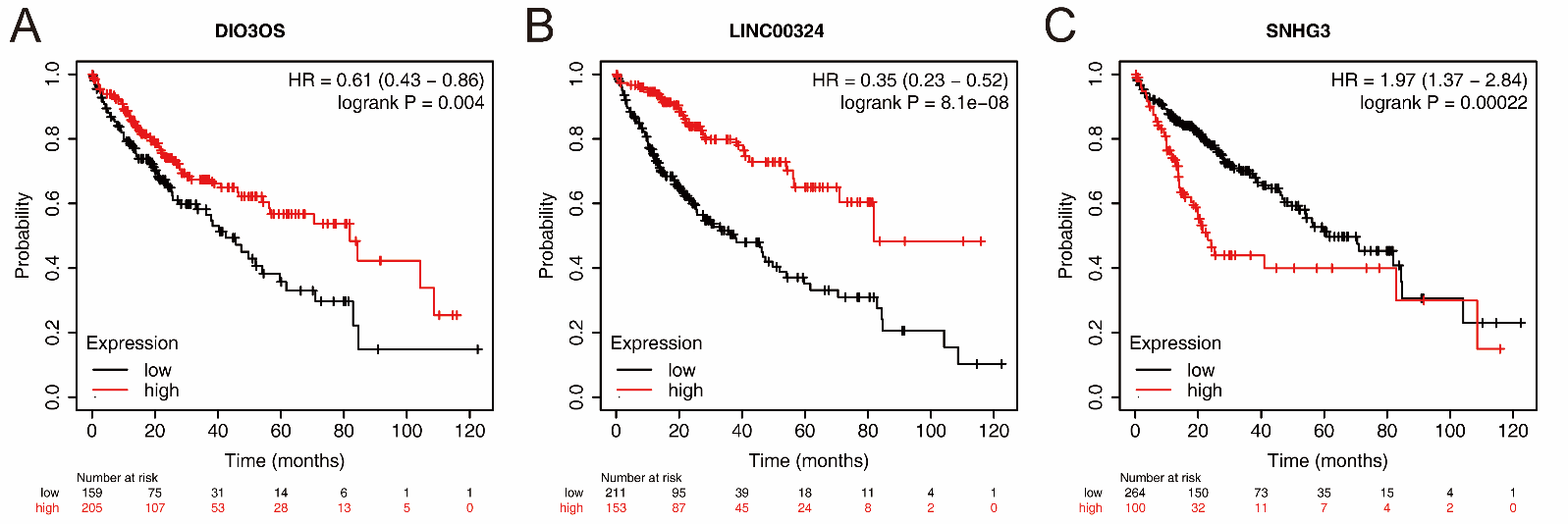

Supplement: Supplementary file 4 — Additional file 4: Figure S3. The expression of (A) DIO3OS (B) LINC00324 and (C) SNHG3 were associated with the OS in LIHC patients in Kaplan Meier Plotter server. [file 12957_2022_2535_MOESM4_ESM.tif]
